# Influence of furfural on the physiology of *Acinetobacter baylyi* ADP1

**DOI:** 10.1093/femsle/fnae059

**Published:** 2024-07-29

**Authors:** José Eduardo Arteaga, Ernesto Rivera-Becerril, Sylvie Le Borgne, Juan-Carlos Sigala

**Affiliations:** Posgrado en Ciencias Naturales e Ingeniería, Universidad Autónoma Metropolitana, Unidad Cuajimalpa. Av. Vasco de Quiroga 4871, Col. Santa Fe Cuajimalpa, Delegación Cuajimalpa de Morelos, Ciudad de México, C.P. 05348, México; Departamento de Ciencias Naturales, Universidad Autónoma Metropolitana, Unidad Cuajimalpa. Av. Vasco de Quiroga 4871, Col. Santa Fe Cuajimalpa, Delegación Cuajimalpa de Morelos, Ciudad de México, C.P. 05348, México; Departamento de Procesos y Tecnología, Universidad Autónoma Metropolitana, Unidad Cuajimalpa. Av. Vasco de Quiroga 4871, Col. Santa Fe Cuajimalpa, Delegación Cuajimalpa de Morelos, Ciudad de México, C.P. 05348, México; Departamento de Procesos y Tecnología, Universidad Autónoma Metropolitana, Unidad Cuajimalpa. Av. Vasco de Quiroga 4871, Col. Santa Fe Cuajimalpa, Delegación Cuajimalpa de Morelos, Ciudad de México, C.P. 05348, México

**Keywords:** *Acinetobacter*, furfural, RT-qPCR, lignocellulosic biomass, acetate metabolism, difurfuryl ether

## Abstract

Pretreatment of lignocellulosic biomass produces growth inhibitory substances such as furfural which is toxic to microorganisms. *Acinetobacter baylyi* ADP1 cannot use furfural as a carbon source, instead it biotransforms this compound into difurfuryl ether using the reduced nicotinamide adenine dinucleotide (NADH)-dependent dehydrogenases AreB and FrmA during aerobic acetate catabolism. However, NADH consumption for furfural biotransformation compromises aerobic growth of *A. baylyi* ADP1. Depending on the growth phase, several genes related to acetate catabolism and oxidative phosphorylation changed their expression indicating that central metabolic pathways were affected by the presence of furfural. During the exponential growth phase, reactions involved in the formation of reduced nicotinamide adenine dinucleotide phosphate (NADPH) (*icd* gene) and NADH (*sfcA* gene) were preferred when furfural was present. Therefore a higher NADH and NADPH production might support furfural biotransformation and biomass production, respectively. In contrast, in the stationary growth phase genes of the glyoxylate shunt were overexpressed probably to save carbon compounds for biomass formation, and only NADH regeneration was appreciated. Finally, disruption of the *frmA* or *areB* gene in *A. baylyi* ADP1 led to a decrease in growth adaptation and in the capacity to biotransform furfural. The characterization of this physiological behavior clarifies the impact of furfural in *Acinetobacter* metabolism.

## Teaser

*Acinetobacterbaylyi* ADP1 decreases its growth and modifies the expression of certain metabolic genes when grown in the presence of furfural.

## Introduction

The development of alternative energies to petroleum is needed to avoid pollution and due to the eventual exhaustion of this fossil resource (Isikgor and Becer 2015, Zoghlami and Paës 2019). Lignocellulosic biomass can be an excellent option for it (Zoghlami and Paës 2019). Pentoses and hexoses contained in lignocellulosic biomass can be used as a carbon source for microorganisms to produce a variety of compounds such as biofuel, biochemicals and biomaterials (Isikgor and Becer 2015). Lignocellulosic biomass must be pretreated to release the fermentable sugars it contains. During one of the most common pretreatments, i.e. dilute acid hydrolysis at high temperatures, aldehydes such as furan-2-carbaldehyde (furfural) and 5-(hydroxymethyl)furan-2-carbaldehyde (hydroxymethyl furfural, HMF) are formed from xylose and glucose degradation, respectively (Palmqvist and Hahn-Hägerdal 2000a, Allen et al. 2010). These aldehydes inhibit the growth of those microorganisms employed in the fermentation of lignocellulosic biomass hydrolysates (Palmqvist and Hahn-Hägerdal 2000b).

Overall, aldehydes are known for their toxic effects on microorganisms by damaging proteins, lipids, and nucleic acids and hampering the production of targeted compounds (Zaldivar et al. 1999, Mills et al. 2009, Jayakody and Jin 2021). Furan aldehydes can be found at concentrations ≤5 g L^−1^ in lignocellulosic hydrolysates (Mills et al. 2009). The mechanisms of furfural and HMF toxicity are mainly by cell membrane disruption, accumulation of reactive oxygen species, enzyme inhibition and damage, breaks or mutations of deoxyribonucleic acid (DNA), being furfural more toxic than HMF (Zaldivar et al. 1999, Mills et al. 2009, Allen et al. 2010, Willson et al. 2022). Furfural also has a synergistic toxic effect with other lignocellulosic-derived inhibitors such as acetic acid (Zaldivar and Ingram 1999, Mills et al. 2009).

In *Escherichia coli*, furfural did not seem to cause membrane disruption despite its hydrophobicity (log P_octanol/water_ = 0.41). However, derivatives such as furan-2-ylmethanol and furan-2-carboxylic acid did (Zaldivar et al. 1999, 2000, Mills et al. 2009). Membrane damage was found in *Saccharomyces cerevisiae* when cells were exposed to furfural (Allen et al. 2010). Regarding enzyme inhibition, several authors have reported that the competition for NAD(P)H cofactors by oxidoreductase enzymes used in furfural detoxification is detrimental to growth (Miller et al. 2009a, b , Arteaga et al. 2021). In addition, protein damage might be attributed to the reaction between their nucleophilic sites and the furan aldehydes (Kurgan et al. 2019). Moreover, the interaction of furfural with duplex DNA causes single-strand breaks in sites with three or more thymine or adenine bases (Mills et al. 2009) while, in the plasmid pBR322, furfural produced DNA mutations (Mills et al. 2009).

Strategies to overcome the toxicity of furfural and HMF generally rely on the detoxification of lignocellulosic biomass hydrolysates using chemical, physical, or biological methods (Palmqvist and Hahn-Hägerdal 2000a, Koopman et al. 2010). Among the biological methods, microbial degradation of furan aldehydes has been studied showing that redox reactions are the key points of the process (López et al. 2004, Wierckx et al. 2011).

The aerobic bacteria *Cupriavidus basilensis* HMF14 and *Pseudomonas putida* Fu1 can use furfural and HMF as carbon sources through the use of oxidoreductase enzymes (Koenig and Andreesen 1990, Koopman et al. 2010). Under aerobic conditions, furans aldehydes are first oxidized to furan-2-carboxylic acid and then metabolized towards 2-oxopentanedioic acid which feeds the tricarboxylic acid cycle (TCA) cycle to produce energy and carbon building blocks (Trudgill 1969, Koenig and Andreesen 1990, Koopman et al. 2010, Nieves et al. 2015). The use of furan aldehydes as carbon sources might be due to the presence of oxygen-dependent oxidoreductases, which can limit the process in anaerobic microorganisms (Koopman et al. 2010, Ran et al. 2014). However, it has been demonstrated that *Desulfovibrio furfuralis*, a strictly anaerobic bacterium, can also use furfural as the sole carbon source yielding acetate. In this organism furfural is transformed into 5-oxo-4,5-dihydrofuran-2-carboxylic acid, which can be hydrolyzed or decarboxylated to 4-oxobutanoic acid, transformed into acetyl-CoA and finally into acetate (Folkerts et al. 1989).

Model microorganisms such as *S. cerevisiae* and *E. coli*, which do not have furan aldehyde oxidative degradation pathways and cannot use these compounds as carbon sources, are known to reduce furan aldehydes through the use of NAD(P)H-dependent oxidoreductases to furan alcohols, which are less toxic and not metabolized (Gutiérrez et al. 2002, Nieves et al. 2015).

In *S. cerevisiae*, it has been demonstrated that furan aldehydes reduction to the corresponding alcohols is catalyzed by NAD(P)H-dependent oxidoreductases such as aldose reductase (EC 1.1.1.21, GRE3), aldehyde dehydrogenase (EC 1.2.1.4, ALD4), alcohol dehydrogenase (EC 1.1.1.2, ADH6) and alcohol dehydrogenase (EC 1.1.1.2, ADH7) (Almeida et al. 2008, Lewis Liu et al. 2008, Nieves et al. 2015).

Furthermore, it has been shown that several enteric bacteria such as *E. coli, Enterobacter aerogenes, Citrobacter freundii, Klebsiella pneumoniae, Klebsiella oxytoca, Edwardsiella* spp., *Proteus vulgaris* and *Proteus mirabilis* transform furfural and HMF into alcohols under aerobic and anaerobic conditions when carbon sources such as glucose, peptone and yeast extract are used (Boopathy et al. 1993, Gutiérrez et al. 2002).

In *E. coli*, the NADPH-dependent oxidoreductases alcohol dehydrogenase (EC 1.1.1.2, YqhD) and methylglyoxal reductase (EC 1.1.1.274, DkgA) are involved in the reduction of furfural to furan-2-ylmethanol (Miller et al. 2009b, Turner et al. 2011). In addition, *E. coli* LY01 employs a furfural NADPH-dependent reductase to reduce furfural to furan-2-ylmethanol (Gutiérrez et al. 2006).

In a previous study has been reported the use of *Acinetobacter* strains to reduce furfural into the less toxic compound difurfuryl ether when acetate was used as a carbon source. *Acinetobacter baylyi* ADP1 possesses two NAD(P)H-dependent alcohol dehydrogenases (EC 1.1.1.284, FrmA and EC 1.1.1.90, AreB) which are responsible for this biotransformation (Arteaga et al. 2021).

NADPH is the preferential electron donor used in the biosynthesis of most cellular components (Ying 2008, Spaans et al. 2015) while NADH is mainly used as the electron donor in catabolic reactions and for aerobic energy production (Ying 2008, Spaans et al. 2015). Several authors have described that both NADH and NADPH are needed for furfural detoxification (Gutiérrez et al. 2006, Arteaga et al. 2021; Miller et al. 2009b; Mills et al. 2009, Nieves et al. 2015). Therefore, variations in the central carbon metabolism and oxidative phosphorylation might occur in the presence of furfural due to the competition for NAD(P)H cofactors used for the detoxification of this compound and the production of biomass and cellular energy.

For this reason, we evaluated the expression level of central carbon metabolism and oxidative phosphorylation genes to study the influence of furfural biotransformation on the physiology of *A. baylyi* ADP1.

## Materials and methods

### Bacterial strains, growth media, and cultivation conditions

The strain *A. baylyi* ADP1 was kindly donated by Professor Veronique de Berardinis (Genoscope CNS, France).

Cultivations of ADP1 were performed at 30°C in 500 mL quadruple baffled Erlenmeyer flasks containing 50 mL of M9 medium using 4 g L^−1^ acetate as the carbon source. When needed, two pulses of 0.5 g furfural L^−1^ were added with a period of 30 min between them. Inocula and M9 medium were prepared as reported elsewhere (Sigala et al. 2017).

The *A. baylyi* ADP1 Δ*frmA* and *A. baylyi* ADP1 Δ*areB* mutant strains were cultivated as follows. Preinocula was performed in 15 mL conical centrifuge tubes at 30°C in lysogeny broth (LB) medium added with 2 g glucose L^−1^. Inocula were performed in 150 mL quadruple baffled Erlenmeyer flasks at 30°C either in LB broth added with 2 g glucose L^−1^ or in M9 medium containing 3 g acetate L^−1^. Chloramphenicol was added at a final concentration of 20 µg mL^−1^ in mutants preinocula and inocula. Cultivations were performed at 30°C in 250 mL quadruple baffled Erlenmeyer flasks containing 25 mL of either LB broth with 1 g glucose L^−1^ or M9 medium with 2 g acetate L^−1^ and 0.6 g furfural L^−1^.

In all the cultivations, two technical replicates from two independent biological experiments were performed. Additionally, for the quantification of cofactors, an unpaired *t*-test was performed, with a significant difference *P* < .05.

### Analytical methods

Bacterial growth was monitored spectrophotometrically at 600 nm using an Eppendorf BioPhotometer^TM^. The OD_600nm_ was converted to dry cellular weight according to the correlation 1 OD_600nm_ = 0.55 g_DCW_ L^−1^ for *A. baylyi* ADP1 as described (Sigala et al. 2019).

Cofactors quantification was performed with the nicotinamide adenine dinucleotide phosphate / reduced nicotinamide adenine dinucleotide phosphate (NADP/NADPH) (Cat. No. MAK038) and nicotinamide adenine dinucleotide / reduced nicotinamide adenine dinucleotide (NAD/NADH) (Cat. No. MAK037) kits from Sigma-Aldrich (MO, USA) following the manufacturer instructions, sampling at exponential and stationary growth phase 10 min before and after two pulses of 0.5 g furfural L^−1^ were added with a period of 30 min between them.

Furfural was spectrophotometrically determined at 520 nm based on a colorimetric reaction with aniline and chlorane. For this, 500 µL of culture were collected and completed to 2 mL with fresh culture medium and sonicated for 4 cycles of 15 s on and 15 s off in a probe tip sonicator. A volume of 2 mL of ethanol was then added, mixed by vortexing and sonicated into an ultrasonic bath for 5 min. Samples were centrifuged at 10 000 rpm for 5 min. A volume of 1 mL of the obtained supernatant was added to a glass cuvette, followed by 20 µL of aniline and 10 µL of chlorane, mixing by vortex, and incubation at room temperature for 20 min after which the absorbance at 520 nm was determined.

### Ribonucleic acid (RNA) isolation and purification

Cell samples for RNA isolation were collected from cultures in exponential and stationary phases 10 min before and after two pulses of 0.5 g furfural L^−1^. Cell lysis and total RNA isolation and purification were carried out using the RNeasy Mini Kit according to the manufacturer's recommendations (Qiagen, Hilden, Germany). All RNA samples were subjected to DNase treatment using the TURBO DNA-free kit (Ambion, MA, USA). The RNA concentration was determined by UV spectrophotometry in a Nanodrop 2000 spectrophotometer (Thermo Fisher Scientific Inc, MA, USA). The absence of DNA contamination in the RNA samples was confirmed by polymerase chain reaction (PCR) amplification of specific control genes. RNA integrity was evaluated by electrophoretic separation on a microfluidic chip in a 2100 Bioanalyzer using RNA 6000 Nano Reagents Part I and RNA Nano Chips (Agilent, CA, USA). The RNA integrity number (RIN), which gives a numerical assessment of RNA integrity, was automatically calculated by the included 2100 Expert Software (Agilent, CA, USA). All samples had RIN values >8.

### cDNA synthesis and transcriptional analysis

The RevertAid H Minus First Strand cDNA Synthesis Kit was used to synthesize cDNA (Thermo Fisher Scientific Inc, MA, USA). For each reaction, approximately 5 µg of RNA and a mixture of 1 × 10^−5^ M DNA reverse primers (Supplementary Table 1) specific for each analyzed gene were used. The *secA* gene was chosen as the reference gene because its Cq practically remained constant under all the tested conditions. Reverse transcription-quantitative polymerase chain reaction (RT-qPCR) was performed in a 7500 Real-Time PCR System (Applied Biosystems, CA, USA) using a SYBR Green/ROX qPCR Master Mix kit (Thermo Fisher Scientific Inc, MA, USA). The size of all amplimers was 101 bp. For each gene, all experiments were performed in duplicate from two different cultivations, and very similar values were obtained. A non-template control reaction mixture was included for each gene. The 2^−ΔΔCq^ method was used to analyze the data (Schmittgen and Livak 2001). For downregulation, negative values were obtained by 1/2^−ΔΔCq^ for each condition. All RT-qPCR experiments were compliant with the minimum information for publication of quantitative real-time PCR experiments (MIQE) guidelines (Bustin et al. 2009). For each gene analyzed after furfural pulses, the transcriptional level of the same gene analyzed without furfural pulses was considered equal to one and was used as a control to normalize the data.

### Inactivation of frmA and areB genes in *A. baylyi* ADP1

Mutant strains *A. baylyi* ADP1 Δ*frmA* and *A. baylyi* ADP1 Δ*areB* were generated based on the general methodology described (de Berardinis et al. 2008) (Supplementary Fig. 1). To confirm the gene interruptions, chromosomal DNA was extracted from cultures of *A. baylyi* ADP1 Δ*frmA* and *A. baylyi* ADP1 Δ*areB* and used as a template in specific PCR reactions using the primers described in Supplementary Table 2. Single-gene knockout mutants were confirmed by observing the correct size of the two expected PCR products in 1% agarose gels (Supplementary Fig. 2).

## Results and discussion

### Growth of *A. baylyi* ADP1 in minimal medium with furfural

**Figure 1.**
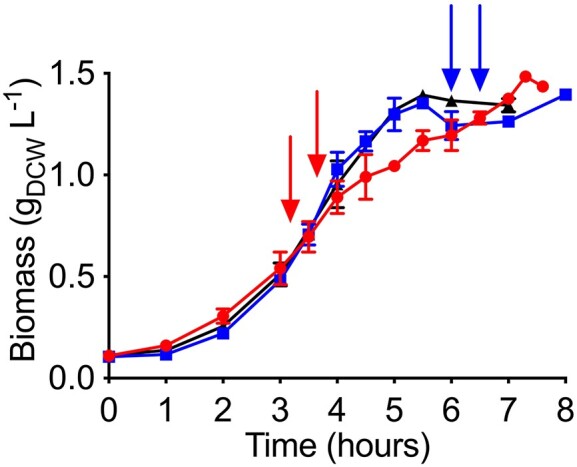
Growth profile of *Acinetobacter baylyi* ADP1 in minimal medium with acetate as carbon source and two pulses of furfural (arrows) either in exponential (red, circles) or in stationary growth phase (blue, squares). A control condition cultivation without furfural is also shown (black, triangles). Error bars represent the experimental error from two independent biological experiments.

The behavior of *A. baylyi* ADP1 after pulses of furfural at exponential and stationary growth phase is shown in Fig. 1. Furfural negatively impacted growth because after its addition the biomass clearly decreased. This could be due to NADH competition (Arteaga et al. 2021) and/or metabolic enzyme damage (Mills et al. 2009). However, after a cell adaptation, growth was recovered. This phenomenon occurred when pulses of furfural were added at exponential or stationary growth phases (Fig. 1).

### Impact of furfural addition in the transcriptional levels of *A. baylyi* ADP1 genes during the exponential growth phase

**Figure 2.**
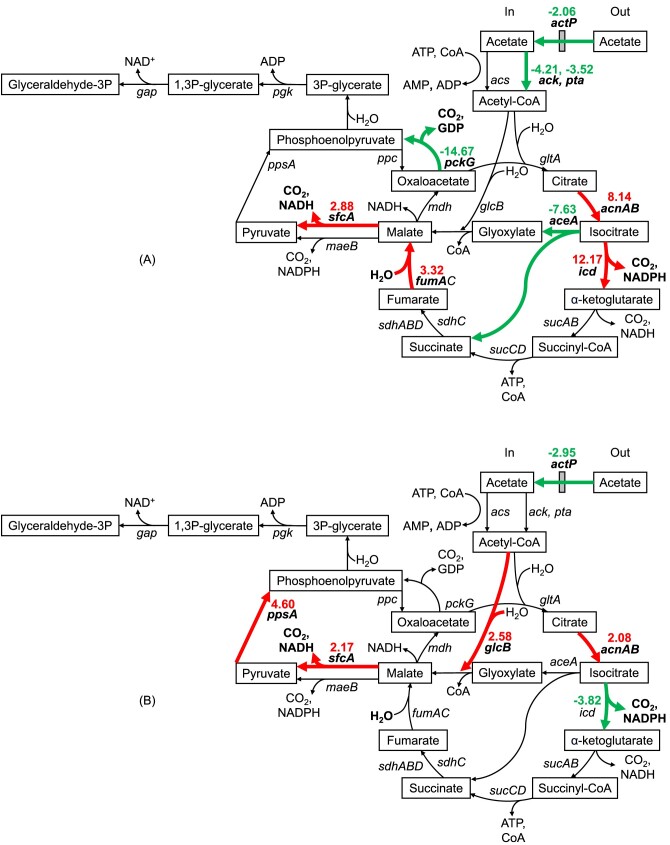
Acetate catabolism of *Acinetobacter baylyi* ADP1 in (A) exponential and (B) stationary growth phase with furfural. Key pathways, metabolites and genes are highlighted. Green, red, and black arrows represent underexpression, overexpression, and no difference of the involved genes, respectively, when furfural is present in comparison with the non-furfural condition.

**Figure 3.**
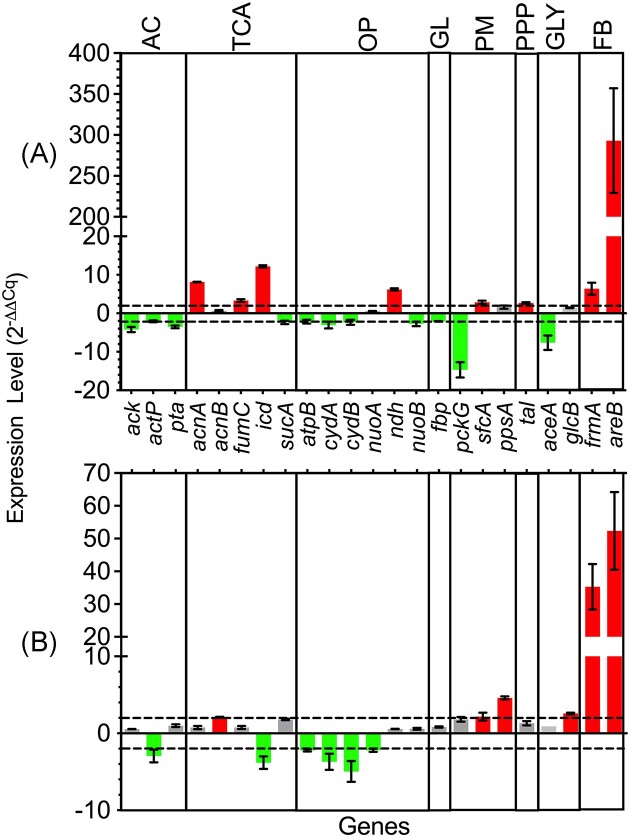
Relative transcription levels of *Acinetobacter baylyi* ADP1 genes in (A) exponential and (B) stationary growth phase during furfural biotransformation. Green, red, and gray bars represent underexpression, overexpression and no difference, respectively, in comparison with the non-furfural condition. Acetate catabolism (AC); tricarboxylic acid cycle (TCA); oxidative phosphorylation (OP); glycolysis/gluconeogenesis (GL); pyruvate metabolism (PM); pentose phosphate pathway (PPP); glyoxylate shunt (GLY); and furfural biotransformation (FB). Error bars represent the experimental error from two independent biological experiments.

The expression levels of genes involved in acetate metabolism and oxidative phosphorylation in *A. baylyi* ADP1 were evaluated after furfural exposure (figs 2A, 3A). Some of the enzymes of these genes require or produce redox cofactors (NAD^+^/NADP^+^). RT-qPCR assay showed that the acetate transporter gene *actP* was underexpressed after furfural addition, suggesting a reduction in acetate transport (Fig. 2A). This could be associated with one of the mechanisms of furfural toxicity which is cell membrane disruption (Mills et al. 2009). The *ack* and *pta* genes were also underexpressed indicating a possible limitation of the Pta-Ack pathway that transforms acetate into acetyl-CoA leading to a decrease in the assimilation of acetate since the Acs pathway was not affected (Fig. 2A).

Under aerobic conditions, the TCA is responsible for the oxidation of acetyl-CoA resulting in the production of intermediates for amino acids, nucleotides, and cofactors synthesis (Han et al. 2008, Kwong et al. 2017). Regarding the TCA, *acnA, icd*, and *fumC* were overexpressed while *aceA* from the glyoxylate shunt was not. Under these conditions, TCA is likely to be preferred over the glyoxylate pathway to generate NADPH for anabolic reactions and NADH for oxidative phosphorylation.

Regarding anaplerotic reactions, the underexpression of *pckG*, whose product transforms oxaloacetate and guanosine triphosphate (GTP) into phosphoenolpyruvate and CO_2_, suggests a flow preference towards the formation of phosphoenolpyruvate via malate and pyruvate with malic enzymes. An increase in the expression of *sfcA* instead of *maeB* demonstrates the priority of generating NADH over NADPH, respectively, when converting malate to pyruvate. In this case, the necessity of NADPH seems to be supplied by the overexpression of *icd* (Fig. 2A). Thus, the NADH generated by the TCA, the malate dehydrogenase (EC 1.1.1.38, SfcA) and the transhydrogenase genes *pntA-1* and *pntA-2*, which are known to increase their expression when furfural is present (Arteaga et al. 2021), seems to be sufficient for oxidative phosphorylation and, at the same time, for the biotransformation of the two pulses of 0.5 g furfural L^−1^ added in this study.

The *ndh* gene showed an increase in its expression level (Fig. 3A). This gene encodes the enzyme NADH: quinone reductase (EC 1.6.5.9, Ndh) which is part of the first complex of the electron transport chain in oxidative phosphorylation. The overexpression of *ndh* may have the purpose of capturing NADH more efficiently given the competition with the enzymes FrmA and AreB which require NADH for furfural reduction. As expected, the genes of these last two enzymes also showed an increase in their expression, mainly *areB*. This is consistent with previous studies (Arteaga et al. 2021). Finally, the oxidative phosphorylation genes *atpB, cydA, cydB*, and *nuoB* were underexpressed, so it could demonstrate that the process is not well-performed in the presence of furfural.

### Impact of furfural addition in the transcriptional levels of *A. baylyi* ADP1 genes during the stationary growth phase

All the cultivations reached the stationary phase upon exhaustion of acetate (data not shown). However, the presence of furfural caused a decrease in the expression of *actP*, but not of the *pta*-*ackA* genes (Fig. 2B). Interestingly, a decreased expression of *icd* suggests the preference for the glyoxylate shunt altogether with the overexpression of *glcB* and *acnB* (Fig. 2B). Furthermore, the expression of *aceA* did not change compared to what occurred in the exponential phase. Under this scenario, the decarboxylations of the lower TCA reactions are prevented at some level in favor of the glyoxylate shunt to save some carbon but with the concomitant decrease in NADPH and NADH. In the early stationary growth phase, biomass is generated at the same rate that it is lysed because the surviving cells use cell debris as a substrate (Navarro Llorens et al. 2010).

In the case of *acnB*, a study has shown that furfural can increase the expression level of this gene in *E. coli* to improve metabolic activity (Miller et al. 2009a). Based on this information, we can deduce that an increment in the expression level of *acnA* and *acnB* in the exponential and stationary phases, respectively, can be attributed in part to the presence of furfural. However, it must be demonstrated if the effect of furfural over the expression levels would be direct or indirect through an intermediary which causes a generalized response to stress as it has been demonstrated in *Zymomonas mobilis* where transcriptional regulators and universal stress genes showed higher expression in presence of furfural (He et al. 2012).

As in the exponential phase, *sfcA* was overexpressed and confirms its role as a generator of NADH via the malate-pyruvate-phosphoenolpyruvate route to support furfural biotransformation. The increase in the expression level of *ppsA* gene demonstrates the preferred formation of phosphoenolpyruvate from pyruvate instead of using the phosphoenolpyruvate carboxykinase (PckG) pathway from oxaloacetate, a phenomenon that was also observed during the exponential growth phase (Fig. 2A).

The genes *frmA* and *areB* were also overexpressed and their products are still responsible for furfural biotransformation at stationary growth phase. It is interesting to note that the overexpression level of *areB* was decreased while that of *frmA* was increased in the stationary compared to the exponential growth phase.

The oxidative phosphorylation genes *atpB, nuoA, cydA*, and *cydB* were underexpressed (Fig. 3B) in the presence of furfural, and the respiration rate decreased in this growth phase (Riedel et al. 2013). This could be also an indirect effect of the diminished levels of NADH caused by the use of this cofactor by the AreB and FrmA enzymes, whose genes were still highly overexpressed (Fig. 3B). Additionally, *ndh* was not overexpressed as in the case of the exponential growth phase, probably because there is no longer a strong NADH competition.

Interestingly, compared to the exponential growth phase fewer genes were affected in their expression levels during the stationary growth phase, which is known to have lower metabolic activity and growth rate (Jaishankar and Srivastava 2017). Consequently, in this growth phase, more NAD(P)H could be available for furfural biotransformation in order to decrease toxicity. In addition, whether in the exponential or stationary growth phase, other genes of the studied pathways, which represent 54% of the total, did not show changes in their expression levels when two pulses of 0.5 g furfural L^−1^ were added (Supplementary Table 3).

This implies that the effect of furfural on the expression of central and energy metabolism genes is specific and is mainly related to oxidative metabolism.

The complete set of genes analyzed by RT-qPCR in exponential and stationary growth phases is shown in the supplementary material.

### Quantification of cofactors in the exponential and stationary growth phases

**Figure 4.**
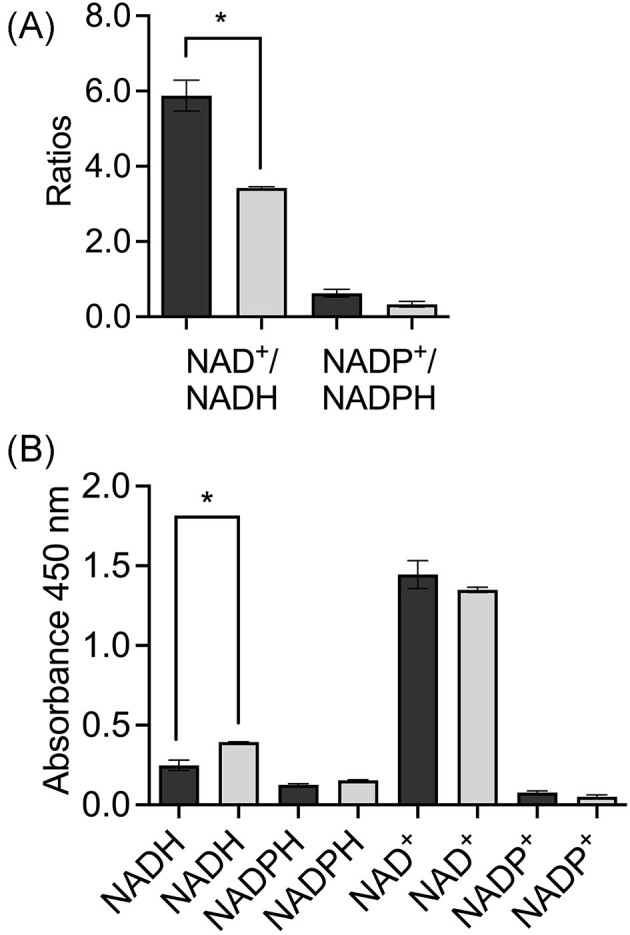
(A) NAD^+^/NADH and NADP^+^/NADPH ratios and (B) relative quantification of reduced and oxidized cofactors in the exponential growth phase of *Acinetobacter baylyi* ADP1. Dark and light bars represent the condition before and after the addition of furfural, respectively. Significant difference (*). Error bars represent the standard error of the mean (SEM) from two independent biological experiments. *P* < .05, degrees of freedom = 2.

The NAD^+^/NADH and NADP^+^/NADPH ratio offers a perspective on the metabolic activity of the cell. Experiments with *Bacillus subtilis* in the presence of colistin showed that an increase in the NAD^+^/NADH ratio stimulated the conversion of NADH to NAD^+^ (Yu et al. 2019). On the contrary, in this work, the presence of furfural showed a decrease in the ratio of both cofactors (Fig. 4A), which suggests the conversion of NAD^+^ to NADH and NADP^+^ to NADPH for furfural reduction in the exponential growth phase. A similar behavior has been shown in *C. glutamicum* and *C. tropicalis* (Tsuge et al. 2014, Wang et al. 2016). However, some microorganisms such as *C. glutamicum* and *S. cerevisiae* are also able to oxidize furfural to 2-furoic acid and consequently, a decrease in reduced cofactors might be attributed due to the changes in the cofactors proportion (Ask et al. 2013, Tsuge et al. 2014). Moreover, it is not clear what the expression level of central carbon metabolism genes was in these microorganisms with furfural and carbon sources to compare with *A. baylyi* ADP1. Overexpression of *sfcA* and *icd* in *A. baylyi* ADP1 could keep the appropriate level of reduced cofactors and avoid their exhaustion. This probably makes a difference between the behavior of *Acinetobacter* and other model microorganisms that are drained in NAD(P)H when furfural is present.

Figure 4B shows the relative level of NADH and NADPH in the cell before and after the addition of furfural. The NADH level slightly increased after the addition of furfural which coincided at least with the overexpression of *sfcA* whose product has NADH as a cofactor like the rest of the reactions that generate this cofactor in the TCA (Fig. 2A). A similar trend is seen with NADPH which is the produced cofactor of isocitrate dehydrogenase (EC 1.1.1.42, Icd) whose gene was also overexpressed (Fig. 3A). The increase in the level of NADH confirmed its demand by FrmA and AreB which are responsible of furfural reduction. There was no significative difference in the levels of NAD^+^ and NADP^+^ (Fig. 4B).

**Figure 5.**
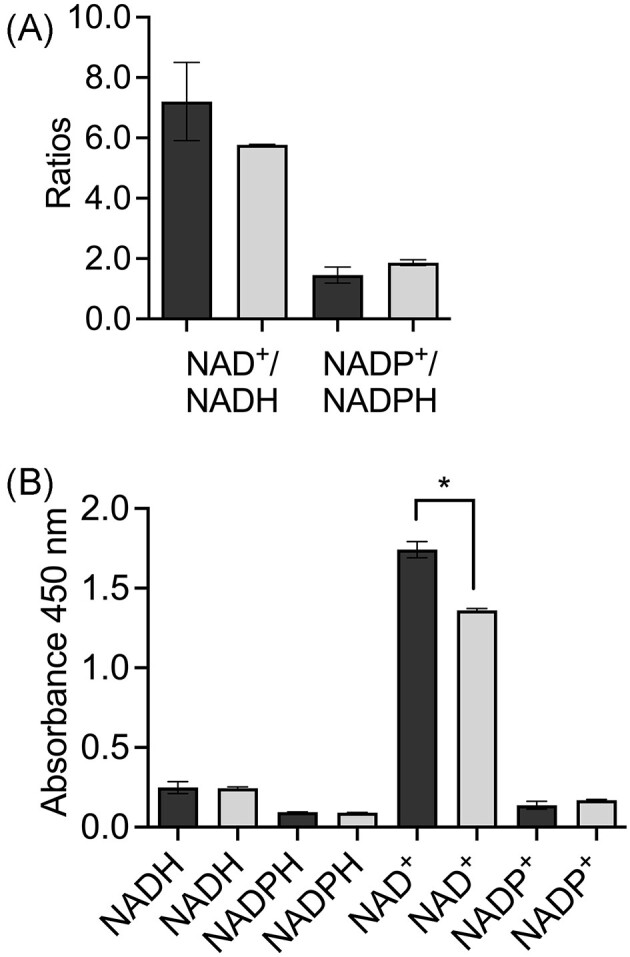
(A) NAD^+^/NADH and NADP^+^/NADPH ratios and (B) relative quantification of reduced and oxidized cofactors in the stationary growth phase of *A. baylyi* ADP1. Dark and light bars represent the condition before and after the addition of furfural, respectively. Significant difference (*). Error bars represent the standard error of the mean (SEM) from two independent biological experiments. *P* < .05, degrees of freedom = 2.

The NAD^+^/NADH and NADP^+^/NADPH ratios did not show significative differences in the stationary growth phase compared to the exponential one (Fig. 5A). However, a downward trend was observed in the NAD^+^/NADH ratio, which would benefit the generation of the NADH required for the biotransformation of furfural. By contrast, the NADP^+^/NADPH ratio showed a slightly upward trend, minimally favoring the generation of NADP^+^.

Figure 5B shows the level of NADH and NADPH present in the cell before and after the addition of furfural in the stationary growth phase. In this case, no changes in their levels were observed. After the addition of furfural, the level of NAD^+^ had a clear decrease (Fig. 5B). As in the exponential phase of growth, a constant regeneration of the reduced form would support the detoxification of furfural. Previous studies showed that, in the presence of furfural, the transhydrogenase genes *pntA-1* and *pntA-2* increased their expression during the stationary growth phase helping furfural reduction (Arteaga et al. 2021).

### Inactivation of frmA and areB in *A. baylyi* ADP1

**Figure 6.**
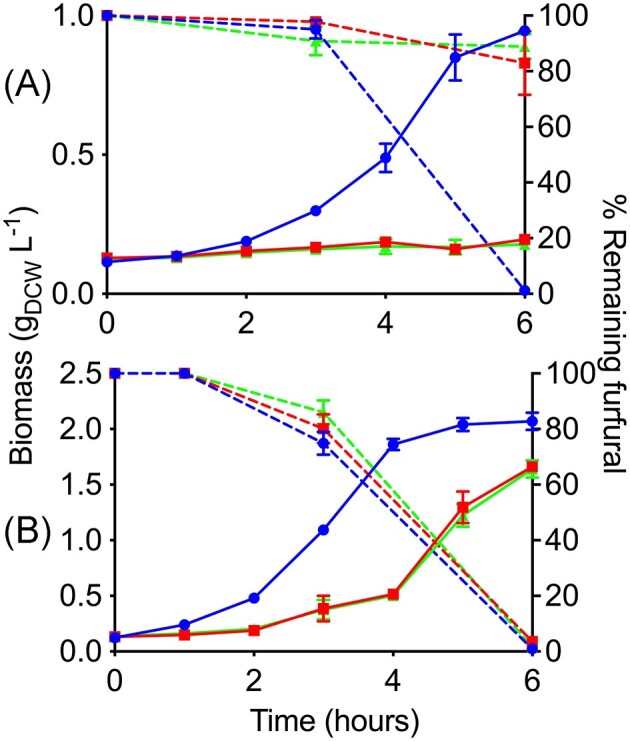
Growth profile (continuous lines) and remaining furfural (discontinuous lines) of *Acinetobacter baylyi* ADP1 (blue, circles), *A. baylyi* ADP1 ∆*frmA* (red, squares) and *A. baylyi* ADP1 ∆*areB* (green, triangles) in (A) minimal medium with acetate and (B) LB-medium with glucose. 0.6 g furfural L^−1^ = 100%. Error bars represent the experimental error from two independent biological experiments.

The growth of *A. baylyi* ADP1 Δ*frmA* and *A. baylyi* ADP1 Δ*areB* was evaluated on M9 medium with furfural and acetate as carbon source (Fig. 6A). Compared to the wild-type strain, the ADP1 mutant strains did not grow after 6 h. In contrast, during this time the wild-type strain reached the stationary growth phase and furfural was completely biotransformed. By the end of the experiment, *A. baylyi* ADP1 Δ*frmA* and *A. baylyi* ADP1 Δ*areB* only biotransformed 17% and 11%, respectively, of the initial furfural (Fig. 6A).

The mutant strains showed a clear deficiency in their ability to reduce furfural. Additionally, when ADP1 and mutants were cultivated in M9 acetate without furfural, the specific growth rate (*µ*) of mutants was affected significantly, decreasing 36% and 26% in Δ*frmA* and Δ*areB*, respectively, in comparison to ADP1 (data not shown). These results suggest FrmA and AreB activities are important for growth and adaptation.

The main consequence of disrupting the *frmA* or *areB* genes was to extend the adaptation phase of bacterial growth in a mineral medium. Both genes are necessary to have a short adaptation phase and to reach maximum biomass in short times to be able to biotransform furfural.

Figure 6B also shows the growth behavior of mutant strains in comparison with the wild type but growing on LB media with glucose and furfural. An affectation was observed in the adaptation time of the mutants. In this rich medium, the mutants could grow and reach the stationary phase, although not as fast as the wild-type strain. *A. baylyi* ADP1 and *A. baylyi* ADP1 ∆*frmA* specific growth rates (*μ*) were 0.70 h^−1^ while that of *A. baylyi* ADP1∆*areB* was 0.68 h^−1^ (a 3% decrease).

Unlike with cultures in the M9 medium, after 6 h in the LB medium, the three strains biotransformed furfural completely despite the fact mutant strains have a longer lag phase (Fig. 6B). Enriched media, such as LB medium, contain a series of non-defined elements and a wide variety of carbon and nitrogen sources (Sezonov et al. 2007, Kim and Kim 2017), as well as a large amount of pre-elaborated building blocks for the formation of macromolecules (Tao et al. 1999), which favors the growth in the mutant strains without compromising energy and reducing power, and compensating the lack of *frmA* or *areB*. In all the above cases, a control sample without bacterial inoculum and under the same experimental conditions confirmed that furfural remains intact without the presence of *Acinetobacter* strains after incubation (data not shown).

The presence of furfural causes key changes in the central carbon metabolism of *A. baylyi* ADP1. During the exponential growth phase, furfural favored the full TCA pathway from Icd over the glyoxylate shunt, with the consequent preferential formation of NADPH and NADH. On the other hand, during the stationary growth phase, the glyoxylate pathway seems to recover its activity. In both growth phases, furfural led to the production of NADH by the malic enzyme SfcA and the malate-pyruvate-phosphoenolpyruvate pathway was preferred. A decrease in the expression levels of most oxidative phosphorylation genes when furfural is present probably decreased the production of energy. Also, cofactors analysis suggests a preference for NADH over NADPH production in the cell as a result of the furfural detoxification by the activity of FrmA and AreB. Finally, the disruption of *frmA* or *areB* in *A. baylyi* ADP1 proved the direct activity of FrmA and AreB in the biotransformation of furfural in this strain.

## Supplementary Material

fnae059_Supplemental_File
