# Supplementary material for: Influence of furfural on the physiology of Acinetobacter baylyi ADP1
Source: FEMS Microbiol Lett. 2024 Jul 29;371:fnae059. doi: 10.1093/femsle/fnae059 (PMC11384913; doi:10.1093/femsle/fnae059)

**Supporting Information.**

Supplementary table 1. Primer sequences used for PCR amplification and RT-qPCR assays in A. baylyi ADP1.

| ORF (gene) | Primer | Sequence (5’ → 3’) |
| --- | --- | --- |
| ACIAD0541  (*ack*) | Forward | GGTACAAGCACCAAGGCAGA |
|  | Reverse | CCAACAAGGTGAGCAGGATTG |
| ACIAD3475  (*acs*) | Forward | TGTGTGGTGTTTGGTGGCTT |
|  | Reverse | CCTCCTCGCATACCTGCATC |
| ACIAD3463  (*actP*) | Forward | ATAACATGGCTGCGGTGCAT |
|  | Reverse | CGCAACAACCGCAAGAATGG |
| ACIAD0540  (*pta*) | Forward | AGCAATACCTTGAGCGTCCC |
|  | Reverse | GAGTGCAGGATACGACGATGT |
| ACIAD1084  (*aceA*) | Forward | CATGGTTTCATCGCGCAACA |
|  | Reverse | AGTGCAGCAACCATCCAACC |
| ACIAD3390  (*acoA*) | Forward | AATTTGCATCGGGCAGCTCA |
|  | Reverse | TTGCGGACGAAGTACCAACT |
| ACIAD3090  (*acnA*) | Forward | TTTCCAGATACGCTGGTGGG |
|  | Reverse | ATGGCTGCTTCTGCTTCGAT |
| ACIAD2395  (*acnB*) | Forward | ACTCACATACGCGTTTCCCA |
|  | Reverse | TTCAGGCATATCGAGTGGCA |
| ACIAD0538  (*fumA*) | Forward | GGTCTTGGCGGTTTGACTAC |
|  | Reverse | GTCGCAGCACAGTTTGGAAT |
| ACIAD1890  (*fumC*) | Forward | ACGAATTGCTTATTCCCGCTG |
|  | Reverse | CTGTAAATGCGTGCGACCAA |
| ACIAD2886  (*gltA*) | Forward | CTACAGTCCGTTTGGCTGGT |
|  | Reverse | TTCGTTTGCACCACCATGAG |
| ACIAD1190  (*icd*) | Forward | CCAGTGGGTGGAGGTATTCG |
|  | Reverse | CTTGGTACACCTTCAAACCAGC |
| ACIAD1187  (*idh*) | Forward | GTGTCAGGTGAAAGACGCAC |
|  | Reverse | GGTACGGGTCAAGCCAGAAA |
| ACIAD3155  (*mdh*) | Forward | CCAGACCTTCCAGCGAAAAAC |
|  | Reverse | ATGTCCGCTACAGCAACACC |
| ACIAD1007  (*mqo*) | Forward | TGTTTATTGGTGCAGGCGGT |
|  | Reverse | CAAGAACACACCGCCCACTG |
| ACIAD2880  (*sdhA*) | Forward | TCGTGGTGAAGGTGCGATTC |
|  | Reverse | GTGAAACAAAGTCACGCGGC |
| ACIAD2882  (*sdhC*) | Forward | CTTGCTCGCTGATCTTGGCTT |
|  | Reverse | CAAAGGCTGCGATGATACCGA |
| ACIAD2876  (*sucA*) | Forward | TGTTCTGTTACCGTCGTCGT |
|  | Reverse | TGTACGCGTTGTTGGCTTTT |
| ACIAD2875  (*sucB*) | Forward | AGCGTTATCCTGCGGTGAAT |
|  | Reverse | CACGAGACCACGATCAGAAGA |
| ACIAD0185  (*atpA*) | Forward | TCCCTGTAGGTCGTGGTCAG |
|  | Reverse | GCCAGAGTGTTTCTGAGCGA |
| ACIAD0180  (*atpB*) | Forward | TGTGGATTGGGTGCCGATG |
|  | Reverse | TCGGATCTGTAGACGGAACG |
| ACIAD0187  (*atpD*) | Forward | GCGATGGTGTTGTTCGTACC |
|  | Reverse | GCCAACTGGAACAGAAATCGG |
| ACIAD0182  (*atpE*) | Forward | CACGTCAACCTGAACTTGCTC |
|  | Reverse | GAACAAGCCAATACCGACACC |
| ACIAD2290  (*cydA*) | Forward | TTTAGGCGTCACTTGGCTGG |
|  | Reverse | TAAATGCGGCACCTACAGGG |
| ACIAD2291  (*cydB*) | Forward | TGGTCCTATTCGCGCTGTTT |
|  | Reverse | AATACTCAGAGCCCAGTCCCA |
| ACIAD2425  (*cyoA*) | Forward | CGGTAAATGAAGTACGCTTCCC |
|  | Reverse | CTGACCGCCTAACTGTGGAA |
| ACIAD2426  (*cyoB*) | Forward | TGAGTTTGCTGCTACAGGCT |
|  | Reverse | AGACTTGCAGTGCCCAGATA |
| ACIAD3633  (*ndh*) | Forward | GGCAATCGGGTCTACATCCA |
|  | Reverse | ATTCACGTTGGAAGCGGTCG |
| ACIAD0730  (*nuoA*) | Forward | GTCGGGTGGATGGGCTTTAC |
|  | Reverse | GACGGAGACCAAGTTAAGGCG |
| ACIAD0731  (*nuoB*) | Forward | GGCTGACCTGATGATTGTTGC |
|  | Reverse | TGGAAATAACCCACTTCGGCT |
| ACIAD2881  (*sdhD*) | Forward | CACACGCATGGATCGGCAT |
|  | Reverse | AGCCGATGTAAGAACCAGACG |
| ACIAD2001  (*eno*) | Forward | AAACTGGCTATAAGGCGGGT |
|  | Reverse | ATTGCCTTCACCTGCCAAGA |
| ACIAD1255  *(epd*) | Forward | GTGTCATGTACCACACAGGC |
|  | Reverse | GCAGTCACGGCATGAATCTC |
| ACIAD2625  (*fbp*) | Forward | TGGGAAGAACCAGTAAAACGCT |
|  | Reverse | GCCACCATACATGCTACCCA |
| ACIAD1925  (*fda*) | Forward | TAGCTCGGTGATGATGGACG |
|  | Reverse | GTGCCATAGCCACTACACGA |
| ACIAD2565  (*gap*) | Forward | CGTGTTGTGTTGACTGCACC |
|  | Reverse | GCCGCAGAGATGATTTTGTCTT |
| ACIAD0101  (*pgi*) | Forward | CGCAATGGGCAAAAAGTGGA |
|  | Reverse | GTCCCTTGATGCAAAAGCTGA |
| ACIAD1927  (*pgk*) | Forward | GTGCTGCTCGTTATGCCAAA |
|  | Reverse | CACCATTGGCTTTTCAGGGG |
| ACIAD2287  (*maeB*) | Forward | ATCAGCCTCCTTTGGTCGTG |
|  | Reverse | CAGAATCCATCGCTGCCTGA |
| ACIAD2842  (*pckG*) | Forward | AGCAGCGACTATGGGTTCTG |
|  | Reverse | TGTTGTAACCCGCGAAAGGT |
| ACIAD3627  *(ppc)* | Forward | CCCTGTTTCATGGTCGTGGT |
|  | Reverse | ACGGATTGCGCCAGAAATTG |
| ACIAD2423  (*ppsA*) | Forward | GCACGTCCTGAAACCGTAAA |
|  | Reverse | CCGATTGAGCGACCTTCACA |
| ACIAD0166  (*sfcA*) | Forward | TTGGGTCTAGGCGATCAGGG |
|  | Reverse | GGCAATGTATAGGCAGGGCT |
| ACIAD0685  (*rpe*) | Forward | TGGTGACTTTCTGGATGCCG |
|  | Reverse | TCGCACCACCAGCCTTAATC |
| ACIAD1358  (*rpiA*) | Forward | GGGTAGAGCAGTTAGGTCGC |
|  | Reverse | GGGATCTCCACCTAAACTCACA |
| ACIAD1980  (*talA*) | Forward | AACTTGACTTTGCTGTTCGGC |
|  | Reverse | TTGTACCAGTCGAGGATACGC |
| ACIAD2035  (*tkt*) | Forward | CGTGGTACGCCAAACCTCAA |
|  | Reverse | AGCGCTGTTGGACCATCTTT |
| ACIAD2335  (*glcB*) | Forward | CGTAAGCAAAGAGCAGGTTGA |
|  | Reverse | AACCAGGAGACATCGGGGTA |
| ACIAD1429  (*areB*) | Forward | TACGCCATCAGCCGTGAAAA |
|  | Reverse | GCACCTGCACCAGTTTGTATG |
| ACIAD1879  (*frmA*) | Forward | GCGTTCGGCATTAGAGTGTG |
|  | Reverse | CGAGCTGAAAAGGTCGAGTTG |

Supplementary table 2. Primer sequences used for gene inactivation in A. baylyi ADP1.

| DNA template | Primer | Sequence (5’ → 3’) |
| --- | --- | --- |
| *frmA* gene from chromosomal DNA | P3A | GCCCAGGTCAACCTTTACAGA |
|  | P4 P1’A | TACCGTCGACCTCGAGAAGTGAACGGAAACACACAAGTTGG |
|  | P5 P2’A | CGGCTAACGTTCTAGCATGCTGTGAATGTTATGCGTTCGG |
|  | P6A | ACGCTACGAATAGACTTACCTTCAT |
| *areB* gene from chromosomal DNA | P3B | CAGTGACCGAATGCAAAGG |
|  | P4 P1’B | TACCGTCGACCTCGAGAAGTCCTGACAGTAGGCTGGTTGTC |
|  | P5 P2’B | CGGCTAACGTTCTAGCATGCTGGACGCCCAGAAATTTTGA |
|  | P6B | GGTTTGAGCGTGATGCCTT |
| pLoxCat2/Cm | P1 | ACTTCTCGAGGTCGACGGTA |
|  | P2 | GCATGCTAGAACGTTAGCCG |
|  | S1 | AGATGTGGCGTGTTACGGTG |
|  | S2 | TCCAGCTGAACGGTCTGGTTA |

Supplementary table 3. Relative transcription values determined by RT-qPCR for A. baylyi ADP1. Positive (red) and negatives (green) values indicate overexpression or underexpression of the associated gene in ADP1 with furfural compared with that of ADP1 without furfural. The results were the average of at least three measurements of the RT-qPCR expression values for each gene. The cDNA was obtained from two independent cultivations. The RT-qPCR expression values obtained for each gene differ <30%.

| Metabolic Pathway | ORF | Gene | Relative expression level in exponential growth phase | Relative expression level in stationary growth phase |
| --- | --- | --- | --- | --- |
| Acetate metabolism | ACIAD0541 | *ack* | -4.10 | 0.55 |
|  | ACIAD3475 | *acs* | 1.76 | 0.54 |
|  | ACIAD3463 | *actP* | -2.04 | -2.95 |
|  | ACIAD0540 | *pta* | -3.48 | 0.98 |
| Tricarboxylic acid cycle | ACIAD3390 | *acoA* | 0.93 | 0.53 |
|  | ACIAD3090 | *acnA* | 8.14 | 0.75 |
|  | ACIAD2395 | *acnB* | 0.67 | 2.08 |
|  | ACIAD0538 | *fumA* | 0.69 | 1.01 |
|  | ACIAD1890 | *fumC* | 3.32 | 0.75 |
|  | ACIAD2886 | *gltA* | 0.97 | 1.02 |
|  | ACIAD1190 | *icd* | 12.17 | -3.64 |
|  | ACIAD1187 | *idh* | 0.83 | 0.88 |
|  | ACIAD3155 | *mdh* | 1.06 | 0.73 |
|  | ACIAD1007 | *mqo* | 1.59 | 1.29 |
|  | ACIAD2880 | *sdhA* | 0.76 | 1.74 |
|  | ACIAD2882 | *sdhC* | 0.54 | 0.66 |
|  | ACIAD2876 | *sucA* | -2.27 | 1.81 |
|  | ACIAD2875 | *sucB* | 0.62 | 1.50 |
| Oxidative phosphorylation | ACIAD0185 | *atpA* | 0.85 | 1.02 |
|  | ACIAD0180 | *atpB* | -2.06 | -2.19 |
|  | ACIAD0187 | *atpD* | 0.80 | 1.14 |
|  | ACIAD0182 | *atpE* | 0.59 | 0.58 |
|  | ACIAD2290 | *cydA* | -2.80 | -3.69 |
|  | ACIAD2291 | *cydB* | -2.14 | -4.96 |
|  | ACIAD2425 | *cyoA* | 0.77 | 0.54 |
|  | ACIAD2426 | *cyoB* | 0.96 | 0.94 |
|  | ACIAD3633 | *ndh* | 6.18 | 0.55 |
|  | ACIAD0730 | *nuoA* | 0.54 | -2.19 |
|  | ACIAD0731 | *nuoB* | -2.65 | 0.58 |
|  | ACIAD2881 | *sdhD* | 0.64 | 1.11 |
| Glycolysis/  Gluconeogenesis | ACIAD2001 | *eno* | 1.10 | 1.10 |
|  | ACIAD1255 | *epd* | 1.47 | 1.16 |
|  | ACIAD2625 | *fbp* | -2.07 | 0.83 |
|  | ACIAD1925 | *fda* | 1.11 | 1.88 |
|  | ACIAD2565 | *gap* | 0.76 | 1.03 |
|  | ACIAD0101 | *pgi* | 1.35 | 1.52 |
|  | ACIAD1927 | *pgk* | 0.77 | 0.96 |
| Pyruvate catabolism | ACIAD2287 | *maeB* | 0.94 | 0.61 |
|  | ACIAD2842 | *pckG* | -14.41 | 1.81 |
|  | ACIAD3627 | *ppc* | 1.75 | 1.62 |
|  | ACIAD2423 | *ppsA* | 1.62 | 4.61 |
|  | ACIAD0166 | *sfcA* | 2.88 | 2.17 |
| Pentose phosphate | ACIAD0685 | *rpe* | 1.30 | 1.27 |
|  | ACIAD1358 | *rpiA* | 1.42 | 1.07 |
|  | ACIAD1980 | *talA* | 2.71 | 1.31 |
|  | ACIAD2035 | *tkt* | 0.88 | 0.75 |
| Glyoxylate shunt | ACIAD2335 | *glcB* | 1.43 | 2.58 |
|  | ACIAD1084 | *aceA* | -7.12 | 0.89 |
| Furfural biotransformation | ACIAD1429 | *areB* | 293.09 | 52.36 |
|  | ACIAD1879 | *frmA* | 6.39 | 35.28 |

Supplementary figure 1. Gene interruption strategy involves amplifying a chloramphenicol integration cassette from the artificial vector pLoxCat2 (PCR1) and two ~320 pb regions (R1 and R2) of the target gene from *A. baylyi* ADP1 chromosomal DNA (PCR2 and PCR3). PCR products are spliced by overlap extension PCR and the final product is used to transform *A. baylyi* ADP1 cells.


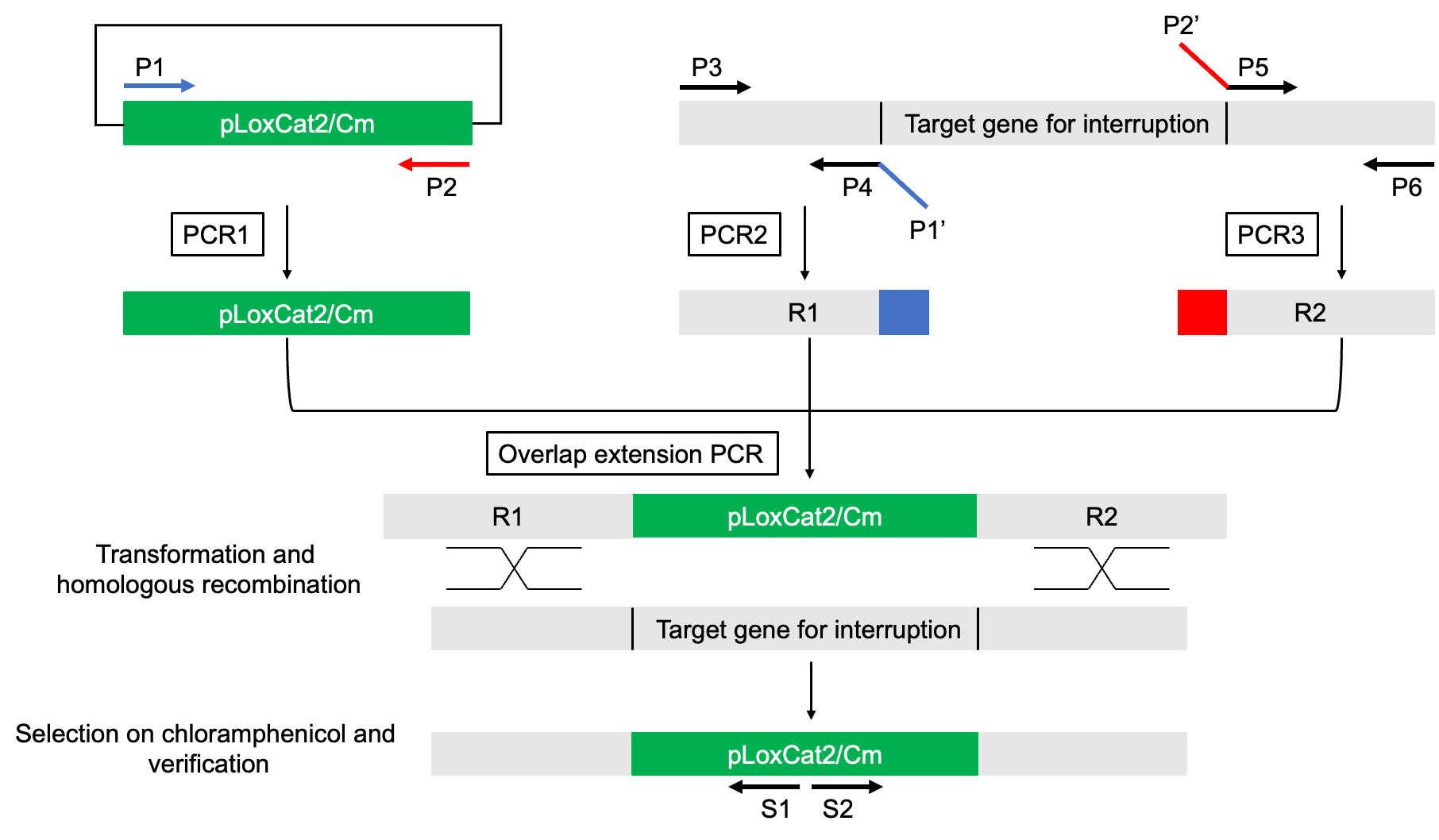


Supplementary figure 2. Expected PCR products from the gene interruption strategy revealed in 1% agarose gel using plasmid and chromosomal DNA from pLoxCat2/Cm and *A. baylyi* ADP1, *A. baylyi* ADP1 Δ*frmA,* and *A. baylyi* ADP1 Δ*areB,* respectively, as template in endpoint PCR reactions with the primers mentioned in supplementary figure 1 and table 2. The first expected PCR product involved a fragment from chloramphenicol resistance cassette: A) ADP1 with S2/P2; B) ADP1 Δ*frmA* with S2/P2; C) ADP1 Δ*areB* with S2/P2; D) pLoxCat2/Cm with S2/P2. The second expected PCR product involved a fragment combining chloramphenicol resistance cassette plus a flanking homologous recombination site: F) ADP1 with P3A/S1; G) ADP1 Δ*frmA* with P3A/S1; H) ADP1 with S2/P6B; I) ADP1 Δ*areB* with S2/P6B. The chromosomal DNA of wild type strain, *A. baylyi* ADP1, was used as a negative control and it showed the absence of the two PCR products above-mentioned validating the successful interruption of *frmA* and *areB* genes.


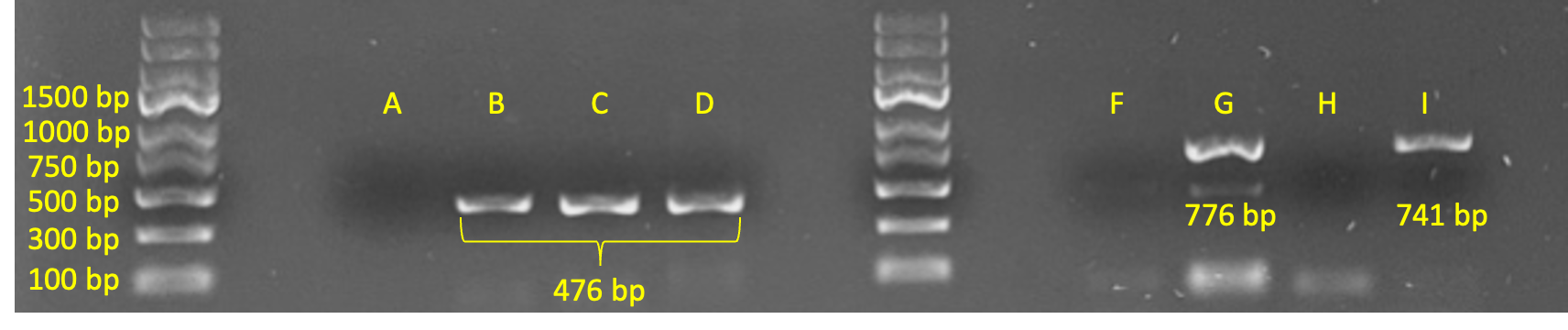

Supplement: fnae059_Supplemental_File [file fnae059_supplemental_file.docx]
